# The sequential binge: a new therapeutic approach for binge eating

**DOI:** 10.1186/2050-2974-3-S1-O57

**Published:** 2015-11-23

**Authors:** Remi Neveu, Guillaume Barbalat, Dorine Neveu, Giorgio Coricelli, Ulrike Schmidt, Alain Nicolas

**Affiliations:** 1 Université de Lyon, Neuroscience Research Center, Lyon, France; 2 Regional Eating Disorders Service, Greenlane Clinical Centre, Auckland, New Zealand; 3 Université Montpellier, Montpellier, France; 4 Interdepartmental Centre for Mind/Brain Sciences (CIMeC), University of Trento, Trento, Italy; 5 Section of Eating Disorders, Institute of Psychiatry, King's College, UK; 6 Hôpital du Vinatier, Bron, France

## Introduction

A significant proportion of eating disorder patients experiencing binge eating do not respond to cognitive behavioral therapies (CBT). Here, we present a new behavioral technique, the sequential binge (SB) that aims at reducing both food intake during binges and daily binge numbers. Specifically, SB replaces the usual pattern of food ingestion during a binge by a repeated monotonous food ingestion sequence interspersed with short incremental pauses. This pattern of ingestion is hypothesised to facilitate boredom towards the ingested foods and promote a sense of control over binge foods.

## Methods

15 eating disorder in-patients with refractory binge eating who were non-responsive to intensive CBT were given SB as an adjunct to their treatment. Patients were followed up for 16 weeks after SB implementation.

## Results

Compared to regular binges, SB was associated with a 44% relative reduction in the planned food intake during binges (p<0.001), a longer refractory period after the binge (median: 48hrs vs. 4hrs, p=0.002), and an average relative reduction of binges by 26% the day after SB (p=0.004).

## Conclusion

This case series shows promising evidence for the use of SB in patients with refractory binge eating. Further evaluation of the use of SB for refractory binge eating in a prospective double-blind clinical study seems justified.

